# Breast Reconstruction in Patients with Prior Breast Augmentation: Searching for the Optimal Reconstructive Option

**DOI:** 10.3390/medicina60101663

**Published:** 2024-10-10

**Authors:** Pasquale Tedeschi, Rossella Elia, Angela Gurrado, Eleonora Nacchiero, Alessia Angelelli, Mario Testini, Giuseppe Giudice, Michele Maruccia

**Affiliations:** 1 Department of Precision and Regenerative Medicine of the Ionian Area, Division of Plastic and Reconstructive Surgery, University of Bari “Aldo Moro”, Piazza Giulio Cesare, 11, 70124 Bari, Italy; 2 Department of Precision and Regenerative Medicine and Jonica Area—(Dimepre-J), U.O.C. of General Surgery “V. Bonomo”, University of Bari “Aldo Moro”, 70121 Bari, Italy

**Keywords:** breast reconstruction, breast augmentation, direct-to-implant, two-stage reconstruction, postoperative complications, patient outcomes

## Abstract

*Background and Objectives*: Breast cancer in patients with prior breast augmentation poses unique challenges for detection, diagnosis, and management. Mastectomy rates are increasing, and patients with prior augmentation often have a lower body mass index, making autologous techniques unsuitable. This study aims to assess the best reconstructive option in patients with a history of subglandular or dual-plane breast augmentation. *Materials and methods*: A prospective analysis was conducted on patients who underwent breast reconstruction after mastectomy. Patients with subglandular or dual-plane breast augmentation were included. Patients were divided into submuscular breast reconstruction (Group 2) or prepectoral breast reconstruction (Group 1) groups. Demographic and surgical data were collected. *Results*: A total of 47 patients were included, with 23 in Group 1 and 24 in Group 2. Complications occurred in 11 patients (23.4%), with significant differences between groups. The most common complication was seroma formation. Implant loss occurred in 4.3% of cases in Group 1, while no implant loss was observed in Group 2. Patient-reported satisfaction scores were similar between groups at 12 months postoperatively. *Conclusions*: Subpectoral breast reconstruction with a tissue expander seems a safer and effective technique for patients with prior breast augmentation. It resulted in fewer complications. This approach should be considered as an option for breast reconstruction after mastectomy in this cohort of patients.

## 1. Introduction

Breast augmentation is the most common surgical esthetic procedure for women worldwide, with nearly 1.9 million procedures performed annually [1]. Regarding breast cancer, there are more than 2.3 million cases each year worldwide, making it the most common cancer among adults [2]. With the increase in breast augmentation procedures, the number of breast cancer patients with a history of prior breast augmentation is also likely increasing. In fact, approximately 1 in 10 of these women may eventually be diagnosed with breast cancer and seek reconstructive options [3]. These patients represent a significant reconstructive challenge because their breast anatomy is altered, but their expectations are very high. Additionally, these patients often prefer implant-based reconstruction (IBR) over autologous reconstruction to maintain their previous esthetic appearance [4,5]. Moreover, patients with a history of prior augmentation often have a lower body mass index (BMI), making them poor candidates for typical autologous techniques.

Therefore, it is crucial to identify key considerations for the detection, diagnosis, and management of breast cancer in women with breast augmentation [6]. While there is no epidemiological evidence linking breast implants to an increased risk of developing breast cancer, concerns remain that implants may delay early mammographic detection, potentially leading to a worse prognosis [7]. Subglandular implants can obscure at least 39% of breast tissue, while subpectoral implants can obscure up to 28% [8,9]. Although evidence is mixed on whether breast cancer presentation differs among women with and without prior breast augmentation, some studies suggest more advanced disease in the augmented cohort, while others show no difference [10,11,12].

The aim of this study was to compare surgical outcomes and complication rates among patients with a history of breast augmentation for cosmetic purposes who underwent mastectomy for breast cancer followed by two-stage submuscular or direct-to-implant prepectoral breast reconstruction.

## 2. Materials and Methods

A prospective analysis was conducted on patients who underwent breast reconstruction after mastectomy at our institution between November 2021 and February 2024. Patients were eligible for inclusion if they had a history of subglandular, submuscular, or dual-plane breast augmentation or augmentation–mastopexy for cosmetic purposes prior to mastectomy and reconstruction. Patients were excluded if they had a history of breast reduction, mastopexy without an implant or any type of breast lipofilling procedure.

Patients were divided into two groups based on the reconstructive technique performed:

- Group 1: Patients undergoing direct-to-implant prepectoral breast reconstruction with a breast implant covered by the acellular dermal matrix.
- Group 2: Patients undergoing two-stage submuscular breast reconstruction.

All patients were assessed intraoperatively for the viability of the mastectomy skin flaps with indocyanine green angiography (ICG). Based on the assessment with ICG, the appropriate reconstructive approach was determined.

Patients who had previously undergone breast augmentation or augmentation mastopexy with subglandular implant placement underwent mastectomy with complete capsulectomy and removal of the previous breast implant. Subsequently, the type of reconstruction was evaluated intraoperatively using ICG.

If the mastectomy flaps exhibited adequate perfusion, a prepectoral direct-to-implant breast reconstruction was chosen, using a breast implant covered by the acellular dermal matrix (ADM). In cases where the mastectomy flaps showed moderate or poor perfusion, a two-stage reconstruction with a tissue expander placed beneath the pectoralis major muscle was performed.

Subsequently, the intraoperative filling of the tissue expander was determined based on the ICG findings. After filling the tissue expander, the viability of the mastectomy flap was reassessed using ICG. If the viability of the mastectomy flap was not clear, the tissue expander was placed empty.

Conversely, patients who had undergone breast augmentation or augmentation mastopexy with the submuscular or dual-plane technique underwent mastectomy with complete capsulectomy and removal of the previous breast implant. Based on the thickness of the mastectomy flaps and the results of the ICG evaluation, the pectoralis major muscle was either reinserted at the costal level for prepectoral reconstruction [13] or used for submuscular reconstruction with a tissue expander.

Patient demographic information and prior surgical history were obtained before enrollment. Additionally, imaging records such as pre- and postoperative MRI scans were evaluated for breast cancer detection and implant position.

Patients’ age, body mass index (BMI), comorbidities, type of breast augmentation or augmentation mastopexy, prior breast implant size, neoadjuvant and adjuvant oncologic treatment, type of mastectomy performed, mastectomy specimen weight, reconstruction technique, implant size, tissue expander size, intraoperative and postoperative TE fill patterns, and final implant size were prospectively recorded.

All patients were followed up with periodic control visits at 1, 3, 6, 12, and 18 months. The occurrence of postoperative complications, including hematoma, seroma, wound dehiscence, infection, major/minor skin necrosis, partial/complete nipple necrosis, and implant loss were noted during each check-up. Long-term complications such as capsular contracture, malposition, and rippling were also assessed during follow-up visits.

The primary outcome measure was the rate of implant-related complications, including wound dehiscence, implant exposure, implant malposition, and rate of mastectomy flap and NAC necrosis. The secondary outcome measures included patient-reported satisfaction with the outcome of the reconstruction. To assess the impact of breast reconstruction on the patients’ health-related quality of life (HRQOL), the BREAST-Q questionnaire reconstructive module was administered to each patient. The preoperative questionnaire was provided to the patients one month prior to surgery, while the postoperative questionnaire was administered one year after the completion of the reconstruction during the programmed clinic visit.

To complement the subjective assessments provided by the BREAST-Q, we incorporated an additional objective evaluation of the esthetic outcomes. Specifically, the Aesthetic Item Scale (AIS) was employed, as described by Dikmans et al. [14]. This method uses five standardized photographs per patient, which are assessed based on five key esthetic parameters: breast volume, shape, symmetry, scars, and the nipple–areola complex (NAC). These criteria were rated on a 5-point Likert scale ranging from “very dissatisfied” to “very satisfied”. The evaluations were performed after the completion of the follow-up by a panel of five independent plastic surgeons specializing in breast reconstruction. These surgeons were blinded to patient details to ensure unbiased assessments, and the photographs were presented on standardized overview sheets to facilitate consistent evaluation across the cohort.

All patients were offered the full range of options for implant-based and autologous reconstruction, including the option of nipple-sparing mastectomy as a treatment for breast cancer. The final choice of reconstructive method was based on discussions between the patient and surgeons. The incision sites were determined and marked jointly by the breast surgeon and plastic surgeon.

The study was approved by the institutional review board and was conducted in accordance with the ethical principles of the Declaration of Helsinki. Informed consent was obtained from all patients.

## 3. Statistical Analysis

Data were analyzed using the Statistical Package for Social Sciences (SPSS) software for Windows, version 23.0 (IBM SPSS, IBM Corp., Armonk, NY, USA). Pearson’s chi-square and Fisher’s test were used for all unadjusted bivariate categorical data comparisons. Student’s *t*-test was used for pairwise continuous data comparisons. Poisson regression was used to estimate the relationship between number of complications and reconstruction procedure type. A *p*-value < 0.05 was considered statistically significant.

## 4. Results

A total of 47 patients were included in the study. Twenty-one patients underwent direct-to-implant prepectoral breast reconstruction with breast implants covered by the acellular dermal matrix (Group 1), and twenty-six patients underwent two-stage submuscular breast reconstruction (Group 2).

In Group 1, 15 patients had previously undergone subglandular breast augmentation, and 6 patients had undergone dual-plane breast augmentation. In Group 2, 13 patients had previously undergone subglandular breast augmentation, 10 had undergone dual-plane breast augmentation, and 3 had undergone total submuscular breast augmentation.

The mean age of the patients was 55.7 years (range 42–68), and the mean body mass index (BMI) was 27.73 kg/m^2^ (range 23.3–30.8). The most common comorbidity was hypertension (38.3%), followed by diabetes mellitus (25.5%) and hyperlipidemia (21.3%). The median follow-up period was 22 months (range 18–26 months).

All patients underwent removal of the implant, capsulectomy, and mastectomy, with 28 (59.6%) undergoing nipple-sparing mastectomy (NSM) and 19 (40.4%) undergoing skin-sparing mastectomy (SSM). The mean mastectomy specimen weight was 296.6 g (range 203–403 g).

The mean preoperative implant size was 365 cc (range 275–450 cc). The mean implant size post-mastectomy in Group 1 was 314.05 cc (range 275–365 cc). The mean tissue expander size in Group 2 was 311.54 cc (range 250–450 cc), with a mean of four fills (range 2–6 fills) required to reach the final implant size. The mean final implant volume in Group 2 was 361.92 cc (range 275–450 cc), representing a mean implant size increase of 16.2% compared to the initial tissue expander volume.

There was no significant difference in the final implant size between the two groups (*p* = 0.212). For patients who underwent two-stage submuscular breast reconstruction, the median time between tissue expander placement and exchange to a permanent implant was 6.64 months (range 5.0–9.0 months). Demographic characteristics and surgical history of the patients are summarized in Table 1.

Postoperative complications were recorded during each postoperative and follow-up visit. A total of 11 complications were observed in 11 patients (23.4%): 8 patients in Group 1 and 3 in Group 2. Table 2 summarizes the incidence and types of complications in each group.

The most common complication observed was seroma formation, which occurred in three patients (6.38%), with two cases in Group 1 and one case in Group 2. No patients (0.0%) developed hematoma. Two patients (4.26%) in Group 1 experienced wound dehiscence. There were two cases (4.26%) of major skin necrosis in Group 1, one case (2.13%) of minor skin necrosis in Group 2, and one case (2.13%) of partial nipple necrosis in Group 2. There were no cases of complete nipple necrosis, implant exposure, explantation, or infection.

In terms of implant loss, three patients (6.38%) in Group 1 experienced complete loss of their implant. These patients underwent explantation and later had successful delayed reconstruction using autologous tissue. None of the patients in Group 2 experienced implant loss.

**Figure 1:**
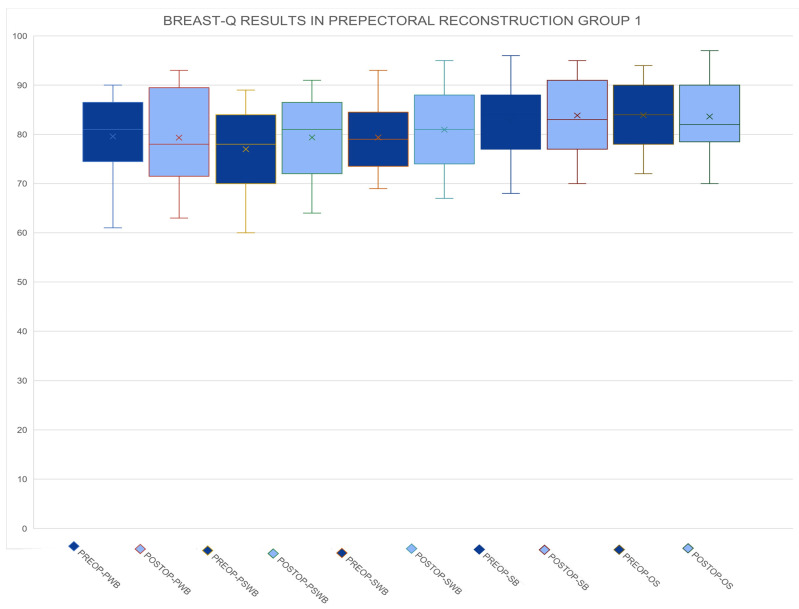
Preoperative and postoperative BREAST-Q scores for Group 1 across all domains, including physical well-being (PWB), psychosocial well-being (PSWB), sexual well-being (SWB), satisfaction with breasts (SB), and overall satisfaction (OS). The comparison highlights changes in patient-reported outcomes before and after surgery.

**Figure 2:**
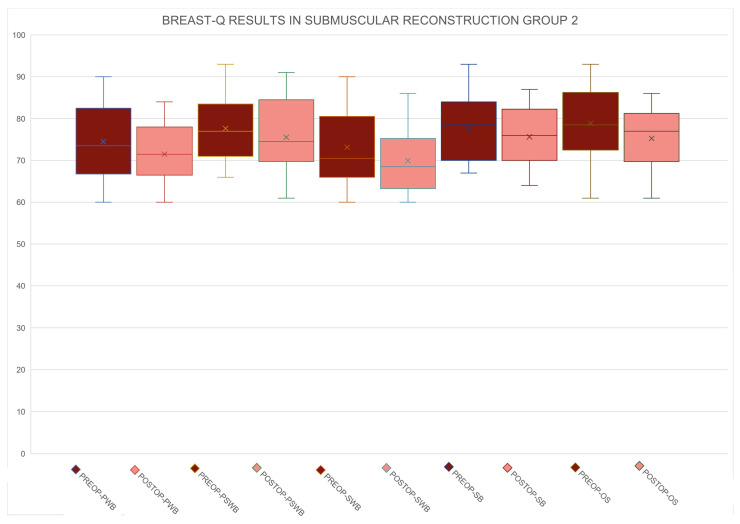
Preoperative and postoperative BREAST-Q scores for Group 2 across all domains, including physical well-being (PWB), psychosocial well-being (PSWB), sexual well-being (SWB), satisfaction with breasts (SB), and overall satisfaction (OS). This comparison demonstrates the impact of the surgical intervention on patient-reported outcomes.

**Figure 3:**
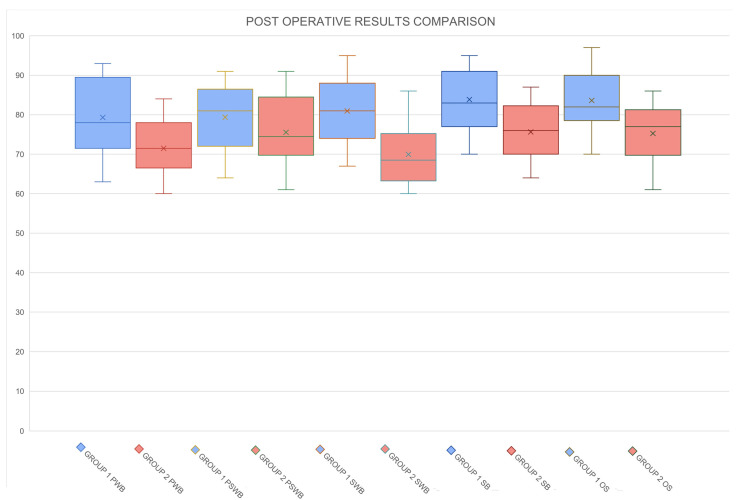
Comparative analysis of postoperative BREAST-Q scores between Group 1 and Group 2 across all domains: physical well-being (PWB), psychosocial well-being (PSWB), sexual well-being (SWB), satisfaction with breasts (SB), and overall satisfaction (OS). The results illustrate the differences in patient-reported outcomes between the two surgical approaches.

The results concerning short-term and long-term postoperative complications are shown in Table 2. Data related to the BREAST-Q scores before surgery and one year after surgery are shown in Table 3 and Table 4 and Figure 1, Figure 2 and Figure 3.

Table 5 shows the mean preoperative and postoperative panel satisfaction with the esthetic results, assessed using the Aesthetic Item Scale (AIS). These data demonstrate a significant improvement in breast volume, shape, and symmetry following reconstruction, as assessed by independent plastic surgeons.

## 5. Discussion

Breast cancer can profoundly impact a woman’s body image, especially for those who have previously undergone breast augmentation for cosmetic reasons. These patients have invested significant time, effort, and resources into enhancing the appearance of their breasts and therefore have high expectations for their breast reconstruction outcomes [15,16,17]. Previous studies have shown that women with a history of breast augmentation are highly demanding after mastectomy and reconstruction [18]. As reported in the study by Clegg et al., 87.5% of augmented patients underwent breast reconstruction with an increased implant volume, 75% with the same implant plane reconstruction, and 68.75% with the same implant-type reconstruction. This means that prior breast augmentation influences the likelihood of undergoing breast reconstruction, with a notable preference for maintaining or increasing implant volume and keeping the reconstruction like the original augmentation in terms of plane and type [4]. The type of reconstruction thus plays a crucial role, but it is important to consider multiple factors that influence the final esthetic outcome. These factors include the type of mastectomy performed, the placement of incisions, and the type of previous augmentation. Each of these elements can significantly impact the overall results and must be carefully evaluated to tailor the reconstruction approach to the patient’s specific needs [19,20,21].

Nipple-sparing mastectomy (NSM) is a safe and esthetically superior approach for appropriate candidates [22,23]. Following NSM, the NAC becomes dependent on the subdermal plexus exclusively, and its viability may be partially compromised by the presence of previous surgical scars. However, patients who have undergone prior breast augmentation or mastopexy are at higher risk for mastectomy flap necrosis, even in the absence of nipple–areolar complex (NAC) preservation, due to compromised tissue perfusion [18,24].

Breast reconstruction goals in patients with prior breast augmentation may differ from those without prior augmentation. Several studies have investigated breast reconstruction in patients with prior cosmetic breast augmentation [25,26,27]. For instance, Frederick et al. found a higher incidence of revision surgeries due to implant-related complications among women with prior augmentation who underwent breast reconstruction post-mastectomy [28]. Other studies report increased risks of complications compared to non-augmentation patients. For example, studies by Alperovich et al. and Sbitany et al. observed an elevated incidence of issues such as infection, capsular contracture, and increased complication rates in patients with a history of augmentation [29,30]. These findings highlight the need for customized preoperative counseling and detailed intraoperative evaluation for women with a history of breast augmentation, addressing their unique concerns and necessitating supplementary preparatory measures to ensure successful reconstruction outcomes.

When considering reconstruction following mastectomy in these patients, it is essential to consider all these factors that can influence the functional and esthetic outcome.

Direct-to-implant prepectoral breast reconstruction is increasingly favored due to its potential to minimize the number of surgeries and deliver superior cosmetic outcomes and patient satisfaction [31,32,33,34]. Prepectoral implant placement avoids disruption of the pectoralis major muscle, reducing the risk of complications associated with submuscular placement, such as animation deformity, chronic pain, and limited upper limb mobility [34,35,36,37]. Moreover, prepectoral placement offers a more natural breast appearance, no upward implant dislocation, and improved esthetics, often eliminating the need for additional contralateral symmetrization procedures [38].

Prepectoral reconstruction, however, may be associated with a higher risk of implant-related complications and delayed wound healing, whereas a subpectoral plane may be associated with a higher risk of implant malposition and animation deformity [39,40].

The objective of the present study was to identify the technique that could offer the best esthetic and functional outcomes for this highly demanding group of patients, while also ensuring the lowest complication rates. A prepectoral breast reconstruction with an acellular dermal matrix (ADM) was offered only after confirming good perfusion of the mastectomy flaps using intraoperative indocyanine green (ICG) angiography. Submuscular reconstruction remained the standard approach for patients with thin mastectomy flaps or compromised flap perfusion, as determined by intraoperative ICG angiography [41]. Although submuscular reconstruction is associated with potential complications such as animation deformity and chronic pain, it provides robust support for the implants and is often necessary when mastectomy flaps are thin or inadequately perfused.

Despite the longer reconstruction times associated with tissue expander use, our study found a lower complication rate in patients undergoing two-stage reconstruction compared to direct-to-implant reconstruction. An overall higher complication rate was observed in the prepectoral reconstruction group (23.4%) compared to the two-stage submuscular reconstruction one (11.5%). The most common complication in Group 1 was seroma formation, followed by wound dehiscence and implant loss. In contrast, Group 2 experienced fewer complications, with only one case of minor skin necrosis and one of partial nipple necrosis. Group 2 did not experience any cases of implant loss. This difference could be explained because previous surgical scars can compromise tissue perfusion, thereby increasing the risk of implant exposure in prepectoral reconstructions (Group 1). Conversely, the presence of a robust muscle layer in submuscular reconstruction (Group 2) may provide better support and perfusion, reducing the risk of complications and implant loss. Notably, there were no cases of hematoma or infection in either group.

Further, while patients in the submuscular group experienced fewer implant-related complications, they also faced issues commonly associated with submuscular placement. Patients who successfully underwent prepectoral reconstruction demonstrated BREAST-Q scores for physical well-being that were comparable to or even better than their baseline scores (mean 80.5 vs. 82.3). In contrast, patients in the submuscular group exhibited significantly lower physical well-being scores post-reconstruction (mean 78.9 vs. 70.2), with these differences being statistically significant (*p*-value < 0.05). This highlights the nuanced outcomes associated with the two reconstruction techniques, emphasizing the trade-offs between complication rates and post-reconstruction quality of life.

The type of initial breast augmentation also plays a crucial role in determining post-reconstruction outcomes. Analyzing the subgroup that underwent submuscular reconstruction starting from a dual-plane breast augmentation, we observed improved physical well-being scores. This improvement is likely because these patients already had a modified pectoral muscle and therefore did not experience a significant change in their physical well-being post-reconstruction. These findings suggest that prior surgical modification of the pectoral muscle may mitigate some of the physical drawbacks associated with submuscular reconstruction.

Furthermore, the satisfaction with the breast and sexual well-being scores were similar in both the prepectoral and submuscular groups (*p* > 0.05), indicating that both techniques can achieve satisfactory esthetic and functional outcomes in these domains. However, overall satisfaction was higher in the prepectoral group, suggesting that this technique may offer a more favorable balance of esthetic outcomes, physical well-being, and patient satisfaction.

Despite the valuable insights provided by our study, there are several limitations that need to be acknowledged. The small sample size limits the generalizability of our findings to a larger population. Furthermore, the relatively short follow-up period prevents a comprehensive evaluation of long-term outcomes and potential complications. Future studies with larger cohorts and longer follow-up durations are necessary to validate these findings and refine the criteria for selecting the optimal reconstructive technique for each patient.

## 6. Conclusions

The choice between prepectoral and submuscular reconstruction should be tailored to the individual patient’s history, anatomical considerations, and personal preferences. While prepectoral reconstruction offers advantages in terms of overall satisfaction and maintenance of physical well-being, it also carries a higher risk of implant-related complications. While our results do not show statistically significant differences, they suggest that submuscular reconstruction, despite its association with lower physical well-being scores, may offer better implant support and reduce the risk of implant loss. Further research with larger sample sizes is necessary to validate these trends.

## Figures and Tables

**Table 1:** Patient demographics and oncologic data.

Characteristic	Direct-to-Implant Reconstruction (*n* = 21)	Two-Stage Breast Reconstruction (*n* = 26)	Total (*n* = 47)
Age, years (mean ± SD)	55.24 ± 8	56.12 ± 7.06	55.7 ± 7.42
Body mass index, kg/m^2^ (mean ± SD)	24.66 ± 1.4	24.8 ± 1.8	24.73 ± 1.62
Comorbidities, *n* (%)			
- Hypertension	10 (47.6)	8 (30.8)	18 (38.3)
- Diabetes mellitus	5 (23.8)	9 (34.6)	14 (25.53)
- Hyperlipidemia	2 (9.52)	2 (7.7)	4 (8.51)
Preoperative implant size, cc (mean ± SD)	364.5 ± 56.1	365.4 ± 54.05	365 ± 54.37
Mastectomy type, *n* (%)			
- NSM	14 (66.7)	14 (56.85)	28 (59.57)
- SSM	7 (33.3)	12 (46.15)	19 (40.43)
Mastectomy specimen weight, g (mean ± SD)	271.43 ± 61.07	316.96 ± 70.66	296.62 ± 69.7
Tissue expander size, cc (mean ± SD)	N/A	311.54 ± 63.73	N/A
Fills required to reach final implant size (mean ± SD)	N/A	4 ± 1.3	N/A
Final implant volume, cc (mean ± SD)	314.05 ± 30.52	361.92 ± 83	340.53 ± 68.73
Access Type for Augmentation, *n* (%)			
- Inframammary fold	8 (38.1)	10 (38.5)	18 (38.3)
- Periareolar	7 (33.3)	9 (34.6)	16 (34.0)
- Inverted-T	6 (28.6)	7 (26.9)	13 (27.7)
Adjuvant/Neoadjuvant Therapies, *n* (%)			
- Neoadjuvant chemotherapy	5 (23.8)	6 (23.1)	11 (23.4)
- Adjuvant chemotherapy	3 (14.3)	4 (15.4)	7 (14.9)
- Radiotherapy	7 (33.3)	8 (30.8)	15 (31.9)

Note: Values are expressed as mean ± standard deviation, median (range), or *n* (%). NSM: nipple-sparing mastectomy; SSM: skin-sparing mastectomy.

**Table 2:** Complications in direct-to-implant and two-stage breast reconstruction groups.

Complication	Group 1 Direct-to-Implant Reconstruction (*n* = 21)	Group 2 Two-Stage Breast Reconstruction (*n* = 26)	Total (*n* = 47)	*p*-Value
Postoperative Complications				
Seroma formation	2 (9.52%)	1 (3.85%)	3 (6.38%)	0.78
Hematoma	0 (0.0%)	0 (0.0%)	0 (0.0%)	
Infection	0 (0.0%)	0 (0.0%)	0 (0.0%)	
Wound dehiscence	2 (9.52%)	0 (0.0%)	2 (4.26%)	0.66
Major skin necrosis	2 (9.52%)	0 (0.0%)	2 (4.26%)	
Minor skin necrosis	0 (0.0%)	1 (3.85%)	1 (2.13%)	0.62
Partial nipple necrosis	0 (0.0%)	1 (3.85%)	1 (2.13%)	0.62
Implant loss	3 (14.3%)	0 (0.0%)	3 (6.38%)	0.27
Long-term Complications				
Malposition	0 (0.0%)	0 (0.0%)	0 (0.0%)	
Capsular contracture	0 (0.0%)	0 (0.0%)	0 (0.0%)	
Rippling	0 (0.0%)	0 (0.0%)	0 (0.0%)	

Note: Values are expressed as mean ± standard deviation, median (range), or *n* (%).

**Table 3:** Preoperative and postoperative BREAST-Q Scores for each domain and each group.

Domain	Group 1 Preoperative (*n* = 21)	Group 1 Postoperative (*n* = 21)	*p*-Value	Group 2 Preoperative (*n* = 26)	Group 2 Postoperative (*n* = 26)	*p*-Value
Physical well-being	75.76 ± 11.04	75.00 ± 8.76	0.73	77.19 ± 8.82	78.69 ± 7.71	0.42
Psychosocial well-being	77.00 ± 8.79	83.62 ± 5.49	0.04	78.65 ± 7.40	72.85 ± 5.45	0.11
Sexual well-being	79.86 ± 7.23	73.86 ± 9.98	0.19	76.15 ± 7.03	74.54 ± 8.44	0.67
Satisfaction with breasts	83.71 ± 7.65	80.24 ± 8.88	0.18	78.12 ± 8.59	75.65 ± 6.52	0.35
Overall satisfaction	83.90 ± 6.77	83.00 ± 8.37	0.89	78.88 ± 8.98	76.88 ± 6.78	0.59

Note: The data are presented as mean ± standard deviation.

**Table 4:** Postoperative BREAST-Q scores for each domain and for each group.

Domain	Group 1 Postoperative (*n* = 21)	Group 2 Postoperative (*n* = 26)	*p*-Value (Group 1 vs. Group 2 Postop)
Physical well-being	75.00 ± 8.76	78.69 ± 7.71	0.15
Psychosocial well-being	83.62 ± 5.49	72.85 ± 5.45	0.00
Sexual well-being	73.86 ± 9.98	74.54 ± 8.44	0.82
Satisfaction with breasts	80.24 ± 8.88	75.65 ± 6.52	0.07
Overall satisfaction	83.00 ± 8.37	76.88 ± 6.78	0.01

Note: The data are presented as mean ± standard deviation.

**Table 5:** Mean preoperative and postoperative panel satisfaction with aesthetic results as assessed by the Aesthetic Item Scale (AIS).

Scores	Preoperative	Postoperative	*p*-Value
Breast Volume	2.27	3.89	<0.05
Breast Shape	1.23	3.21	<0.05
Breast Symmetry	2.01	3.76	<0.05
Breast Scar	3.10	3.40	0.42
Nipple/NAC	2.60	2.90	0.31

Note: Scores were rated on a 5-point Likert scale ranging from 1 (very dissatisfied) to 5 (very satisfied).

## Data Availability

The data supporting the findings of this study are not publicly available due to privacy and ethical restrictions.
